# Elbow Cystic Lymphangioma in an 8-Month-Old Boy

**DOI:** 10.1155/2019/8762614

**Published:** 2019-02-11

**Authors:** Mohammad Jawad H. Rahal, Morad R. Abou Al Ezz, Rabab A. El Hajj, Jad Z. Chokor, Selim M. Nasser, Ramzi C. Moucharafieh

**Affiliations:** ^1^Department of Orthopaedic Surgery and Traumatology, Clemenceau Medical Center Affiliated with Johns Hopkins International, Clemenceau, Beirut, Lebanon; ^2^Department of Orthopaedic Surgery and Traumatology, Faculty of Medical Sciences, Lebanese University, Hadath, Beirut, Lebanon; ^3^Department of Paediatrics, Faculty of Medical Sciences, Lebanese University, Hadath, Beirut, Lebanon; ^4^Department of Pathology, School of Medicine, Lebanese American University, Byblos, Lebanon

## Abstract

Cystic lymphangiomas are benign tumors originating mainly in the head and neck of the pediatric population. The authors report a rare case of cystic lymphangioma in the right elbow of an 8-month-old baby treated successfully by complete surgical resection.

## 1. Introduction

Lymphangiomas are congenital malformations of the lymphatic system accounting for nearly 5% of all benign tumors in children and infants [1].

Composed of cystically dilated lymphatics, they commonly present as a solitary mass particularly in the head and neck areas and rarely in extremities [2]. Because of their size and location, they are sometimes a real therapeutic challenge.

Primary elbow childhood masses have been reported in pilomatrixoma Rosai-Dorfman disease, synovial hemangiomas, fibrosarcoma, and schwannomas [3–8].

We report a very rare case of a large cystic lymphangioma in the elbow of an 8-month-old child.

## 2. Case Report

An 8-month-old baby boy was presented to our clinic with a one month history of right elbow mass. The patient's mother claimed that the child is moving his right upper extremity actively without any limitation. She also reported gradual increase in the mass size in the last month.

The patient is previously healthy without any past medical or surgical history. The patient is a product of full-term pregnancy with no perinatal complications. His developmental history was uneventful with no family history of malignancies.

**Figure 1:**
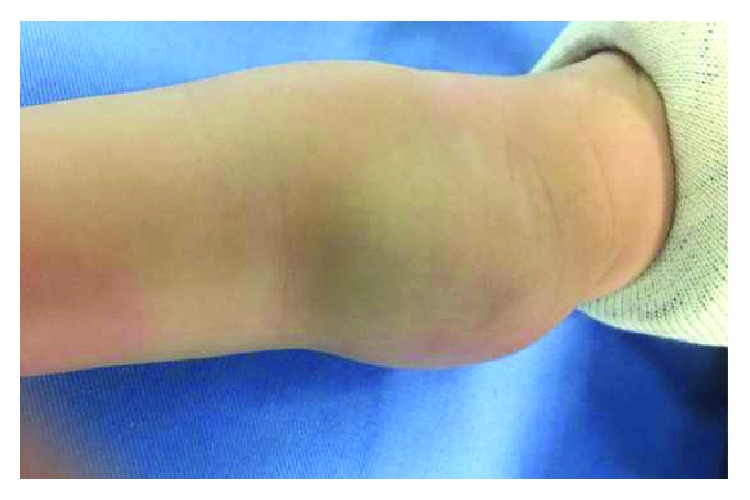
The figure shows appearance of the right elbow mass. The mass is of 3 × 2 × 4 cm.

Physical examination revealed a 3 × 2 × 4 cm mobile soft nontender mass extending from the right proximal ulnar aspect volar surface crossing the elbow crease proximally with no signs of erythema (Figure 1). The full elbow active extension with mild restriction of full flexion is around 15 degrees.

**Figure 2:**
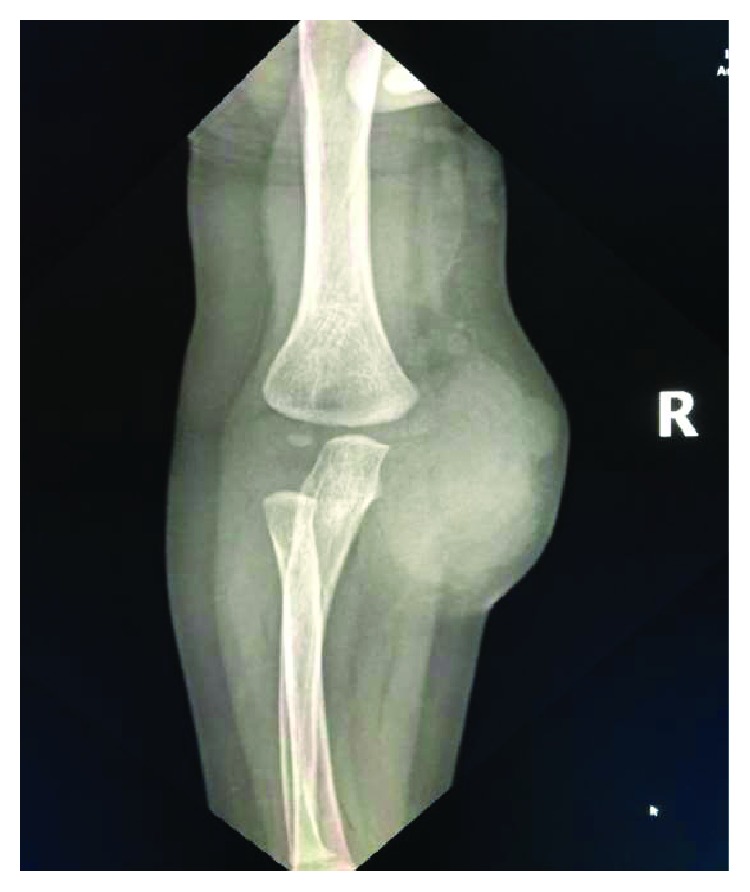
Plain radiograph AP view of the right elbow showed lobular soft tissue swelling suggesting a mass.

Plain radiograph of the right elbow showed a lobular soft tissue swelling (Figure 2) with a normal blood profile (complete blood count, ESR, CRP).

**Figure 3:**
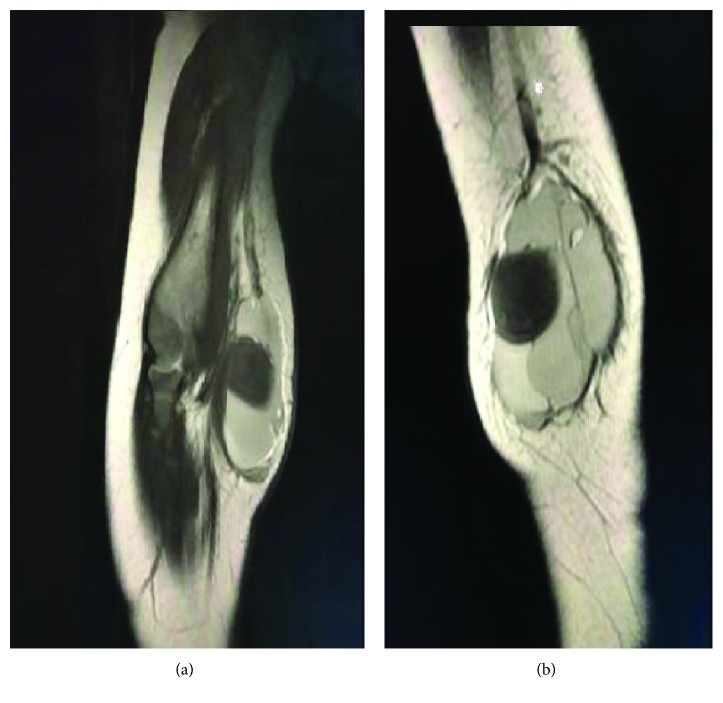
MRI of the right elbow: coronal T2-W1 sagittal T2-W1 sequences of the right elbow shows a lobular juxta articular subcutaneous soft tissue lesion along the medial aspect of the elbow; the lesion appears multiseptated with a predominantly high T2 signal intensity.

**Figure 4:**
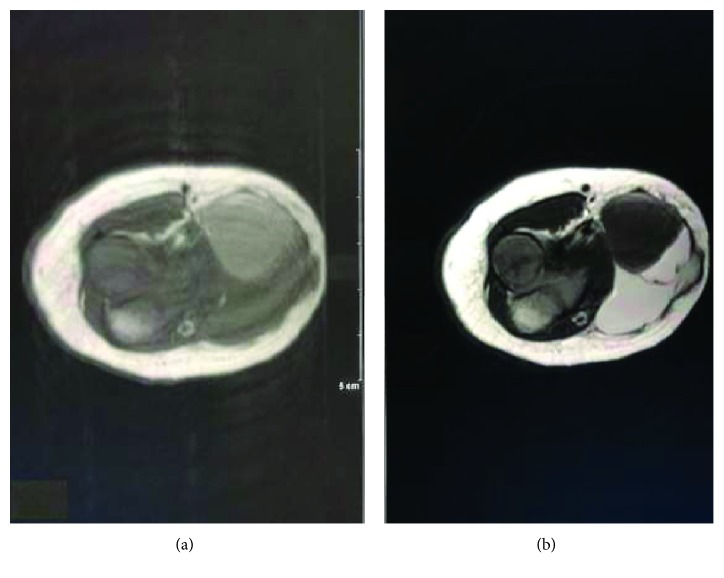
MRI of the right elbow axial T1-W1 (a) and T2-W1 (b) intramass 1.8 × 1.8 × 1.5 cm area with low T2 and high T1 signal intensity.

The MRI of the right elbow revealed a 3.3 × 2 × 4.5 cm (AP, transverse, and CC dimension) lobular juxta articular subcutaneous soft tissue lesion along the medial aspect of the elbow. The lesion appeared multiseptated with a predominantly high T2 signal intensity and contains a 1.8 × 1.8 × 1.5 cm area with low T2 and high T1 signal intensity and no perilesional edema or joint effusion (Figures 3 and 4).

The overall findings were in favor of an endolymphatic malformation. Decision was taken for radical excision.

**Figure 5:**
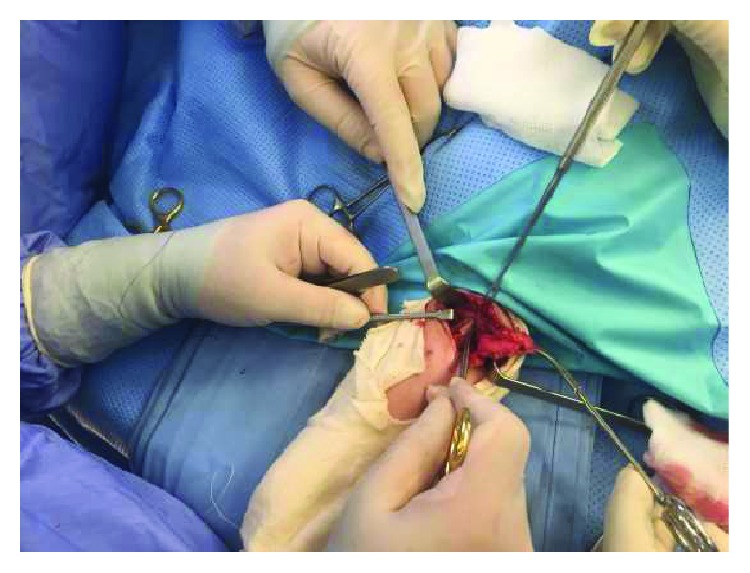
Intra-op capture for the dissection of the mass from the median nerve.

Radical excision was performed under tourniquet control with dissection of the mass from the ulnar, the median nerves, and the antebrachial muscles (Figure 5).

**Figure 6:**
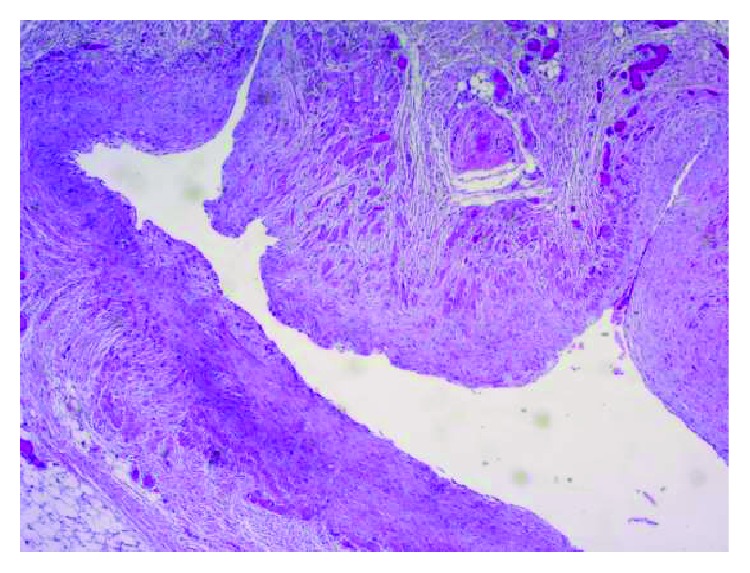
Right elbow lesion showed multiloculated lymphangioma with no evidence of malignancy. The lesion shows vascular spaces with thick vascular walls incorporating adipose tissue and nerve fascicles (H&E, ×200).

Pathology specimen of the tumor showed a multiloculated lymphangioma with no evidence of malignancy (Figure 6). The lesion showed vascular spaces with thick vascular walls incorporating adipose tissue and nerve fascicles. The vascular spaces were lined by regular endothelial cells. Surrounding synovial fluid cytology was negative for malignancy with rare regular cell present.

The patient was discharged day 2 post-op in a good condition.

Follow-up at 2 weeks, 1 month, and 6 months post-op was satisfactory with elbow full range of motion and normal median and ulnar nerve function.

## 3. Discussion

Lymphangioma is a rare benign vascular malformation of the lymphatic system of the childhood composed of cystically dilated lymphatics. They are less common than the tumors of blood vessels (hemangiomas) and commonly present as a mass particularly in the head and neck [2].

Their incidence is reported to be 1.2-1.8 per 1000 of new births [9].

Lymphangiomas are classified as simple or microcystic (formed by lymphatic capillaries), cavernous (formed by bigger lymphatic vessels with a fibrious adventicia), and cystic lymphangiomas (CL) also known as cystic hygromas. Cystic lymphangioma noncommutating masses range from millimeters till centimeters in size [10, 11].

Lymphangiomas appear at birth within the 2nd year of life [12]. 75% of the CL occur in the head and neck and 20% in the axilla [13]. Reported cases site CL in childhood in the abdominal wall with splenic association [14], mediastinum [15], tongue [16], and ovaries and breast [17].

They can occur in adults, usually secondary to local trauma or infections with around 100 reported cases [18].

Actually these tumors are very rare in the extremities [12]. In a recent and large series study, 11 of 186 patients (6%) were presented with lymphangiomas of the upper limbs [2].

Mirza et al. reported 2 cases of cystic lymphangioma of the sternum and a case of upper extremity CL in a 2-month-old male [19]. Furthermore, Greenbaum et al. reported a case of a 2-year-old female with an elbow lymphangioma [20].

They are usually present in the extremities with a vague mass [21] that is either soft or hard induration [22] and often causes pain upon palpation, movement, exercise, and trauma [13, 23, 24] and rarely to cause pain at rest [25].

Hemangiomas are challenging differential for lymphangiomas. They have similar MRI findings in terms of hyperintensity on T2 and hypointensity on T1, but they can be differentiated by the presence of feeding arteries and draining veins found in hemangiomas.

Elbow childhood tumors were reported in different types of masses. Elbow subcutaneous neoplasm cited as pilomatrixoma, which is a rare benign neoplasm that presents as a solitary, hard, and mobile mass [3], was detected in the right forearm and diagnosed as Rosai-Dorfman disease [4]. In addition, a synovial hemangioma of the elbow in an 8-year-old boy was also reported [5].

The pediatric fibrosarcoma of the elbow and the forearm had been also described in the literature [6, 7].

Schwannomas also can present as painless soft tissue mass in the upper extremities but tend to occur more frequently in adults and are often located within a nerve sheath [8].

Malignant tumors such as synovial sarcoma, rhabdomyosarcoma, or lymphoma must be ruled out by the definitive histologic diagnosis.

In our case, malignancy was ruled out by the absence of malignant features (dedifferentiation, mitotic figures, hypercellularity, significant pleomorphism, or necrosis) [26].

Ultrasound is very useful and sensitive in detecting cystic masses. It is superior in terms of compliance and avoidance of the use of anesthetics needed in MRI or CT. It is also useful in assessing postoperative complications and recurrences.

MRI is currently the modality of choice for diagnosis. It is indispensable in the staging of lymphangiomas in addition to its essential role in mapping for surgical excision [27, 28].

Lymphangiomas appear heterogeneous with a low signal intensity on T1-weighted images and high signal intensity on T2-weighted images (fluid-filled cystic spaces [13, 29, 30]. In almost all cases, there exist focal septations and appear as low intensity linear structures of variable thickness that may enhance on gadolinium injection [29, 31].

The natural history of lymphangiomas is variable. Their spontaneous regression is debated and extremely rare.

Malignant transformation is also rare with reported cases in previously irradiated lymphangiomas [32, 33].

Lymphangiomas are usually difficult to manage.

Nonsurgical options include sclerotherapy using intralesional sirolimus [34], bleomycin [35], OK-432—a strain of group A streptococcus [36], doxycycline, ethanol, and hypertonic glucose [37] in adults.

Surgical excision remains the mainstay treatment for soft tissue lymphangiomas [22–24]. Surgical excision is a reliable option in cases of large lymphangiomas or cystic hygromas. However, surgical excision tends to be incomplete due to their diffuse nature [38, 39].

The timing of surgical excision is still controversial. Some authors report that patients presenting with such lesion under 12 months would benefit from surgery between 18 and 24 months [33]. In contrast, others report that resection should be done once such mass is recognized because of its tendency for gradual enlargement [22].

## 4. Conclusion

Cystic lymphangiomas are uncommonly present in the upper extremities of infants. The diagnosis is usually made by magnetic resonance imaging, but histology is indispensable for confirmation. Treatment is surgical as these lesions rarely regress spontaneously especially if they are symptomatic and causing musculoskeletal dysfunction.
